# The Role of Sugarcane Catalase Gene *ScCAT2* in the Defense Response to Pathogen Challenge and Adversity Stress

**DOI:** 10.3390/ijms19092686

**Published:** 2018-09-10

**Authors:** Tingting Sun, Feng Liu, Wenju Wang, Ling Wang, Zhuqing Wang, Jing Li, Youxiong Que, Liping Xu, Yachun Su

**Affiliations:** 1 Key Laboratory of Sugarcane Biology and Genetic Breeding, Ministry of Agriculture, Fujian Agriculture and Forestry University, Fuzhou 350002, China; sunting3221@163.com (T.S.); 18359162091@163.com (F.L.); wwj1470665850@163.com (W.W.); lingw2017@126.com (L.W.); zhuqingemail@163.com (Z.W.); LJ513964@163.com (J.L.); queyouxiong@126.com (Y.Q.); xlpmail@126.com (L.X.); 2 Key Laboratory of Ministry of Education for Genetics, Breeding and Multiple Utilization of Crops, College of Crop Science, Fujian Agriculture and Forestry University, Fuzhou 350002, China

**Keywords:** sugarcane, catalase, *Sporisorium scitamineum*, expression profiles, defense response

## Abstract

Catalases, which consist of multiple structural isoforms, catalyze the decomposition of hydrogen peroxide in cells to prevent membrane lipid peroxidation. In this study, a group II catalase gene *ScCAT2* (GenBank Accession No. KF528830) was isolated from sugarcane genotype Yacheng05-179. *ScCAT2* encoded a predicted protein of 493 amino acid residues, including a catalase active site signature (FARERIPERVVHARGAS) and a heme-ligand signature (RVFAYADTQ). Subcellular localization experiments showed that the ScCAT2 protein was distributed in the cytoplasm, plasma membrane, and nucleus of *Nicotiana benthamiana* epidermal cells. Quantitative real-time polymerase chain reaction (qRT-PCR) analysis indicated that the *ScCAT2* gene was ubiquitously expressed in sugarcane tissues, with expression levels from high to low in stem skin, stem pith, roots, buds, and leaves. *ScCAT2* mRNA expression was upregulated after treatment with abscisic acid (ABA), sodium chloride (NaCl), polyethylene glycol (PEG), and 4 °C low temperature, but downregulated by salicylic acid (SA), methyl jasmonate (MeJA), and copper chloride (CuCl_2_). Moreover, tolerance of *Escherichia coli* Rosetta cells carrying pET-32a-*ScCAT2* was enhanced by NaCl stress, but not by CuCl_2_ stress. *Sporisorium scitamineum* infection of 10 different sugarcane genotypes showed that except for YZ03-258, FN40, and FN39, *ScCAT2* transcript abundance in four smut-resistant cultivars (Yacheng05-179, YZ01-1413, YT96-86, and LC05-136) significantly increased at the early stage (1 day post-inoculation), and was decreased or did not change in the two smut-medium-susceptibility cultivars (ROC22 and GT02-467), and one smut-susceptible cultivar (YZ03-103) from 0 to 3 dpi. Meanwhile, the *N. benthamiana* leaves that transiently overexpressed *ScCAT2* exhibited less severe disease symptoms, more intense 3,3′-diaminobenzidine (DAB) staining, and higher expression levels of tobacco immune-related marker genes than the control after inoculation with tobacco pathogen *Ralstonia solanacearum* or *Fusarium solani* var. *coeruleum*. These results indicate that *ScCAT2* plays a positive role in immune responses during plant–pathogen interactions, as well as in salt, drought, and cold stresses.

## 1. Introduction

Catalase (E.C.1.11.1.6; hydrogen peroxide oxidoreductase; CAT), a heme-containing tetramer enzyme, is one of the major enzyme defense systems widely distributed in living organisms. In higher plants, the main function of catalase is to degrade H_2_O_2_ and contribute to the prevention of oxidative damage caused by various biotic and abiotic stresses [1,2]. To date, *CAT* genes have been cloned and identified in various Graminaceae crops, such as *Oryza sativa* [3], *Zea mays* [4], *Hordeum vulgare* [5], and *Triticum aestivum* [6]. Studies have suggested that CATs are mainly distributed in peroxisomes, glyoxysomes, and the cytoplasm, whereas a small number occur in the mitochondria [7,8].

The expression of catalase genes is regulated by biotic or abiotic stresses [9,10,11,12,13,14,15,16,17,18,19]. Du et al. [9] detected that the expression of the *CAT1*, *CAT2*, and *CAT3* genes, and the enzyme activities of CATs in *Arabidopsis thaliana* could be induced by cold, drought, oxidative stress, salicylic acid (SA), and abscisic acid (ABA). Yong et al. [20] showed that the overexpression of the *IbCAT2* gene of *Ipomoea batatas* confers salt and drought tolerance in *Escherichia coli* and *Saccharomyces cerevisiae*. Purev et al. [17] have reported that *PgCat1* is expressed at different levels in the leaves, stems, roots of *Panax ginseng* seedlings, and is significantly induced by various stresses, including cropper, hyperosmosis, hydrogen peroxide, ABA, SA, jasmonic acid (JA), chilling, and high light irradiances. *Ngcat1* mRNA expression in *Nicotiana glutinosa* is the highest in leaves, and is repressed by treatment with SA [19]. Both the transcripts of *Ngcat1* and its enzyme activity have also been observed in *N. benthamiana* plants eliciting hypersensitive response (HR) during tobacco mosaic virus (TMV) infection [19].

Catalases, which have multiple structural isoforms, are divided into two groups according to molecular structure and amino acid homologies. Group I consists of two 55 kDa and two 59 kDa subunits, with unique amino acid sequences of Ser–Arg–Leu. Group II consists of four 55 kDa subunits with a unique amino acid sequence of Ser–Ser–Ser [5,8]. Different family members of *CATs* exhibit variable expression patterns in plants. Three *CATs* from *A. thaliana* show organ-specific expression, and are differentially expressed in response to various abiotic stresses [9,21]. Similarly, a *Manihot esculenta* Crantz catalase gene, *MecCAT1*, is expressed predominantly in the roots and at very low levels in the leaves, suggesting that *MecCAT1* plays a role in delaying deterioration responses [22].

To date, eight *CAT* genes have been found in *Saccharum* spp. [23,24,25]. We previously described a positive correlation between catalase activity and smut resistance in sugarcane [23]. A plasma membrane and cytoplasm located catalase gene *ScCAT1* (GenBank Accession No. KF664183) was isolated from sugarcane variety Yacheng05-179 after inoculation with smut pathogen *Sporisorium scitamineum* [23]. *ScCAT1* showed a positive response to *S. scitamineum* infection and various abiotic stimuli, such as plant hormone treatment, oxidative stress, heavy metals, and hyperosmotic stresses [23]. A CAT protein sequence (AGT16310.1) from *Saccharum* hybrid cultivar R570 has been submitted to National Center for Biotechnology Information (NCBI), however, its function remains unclear. Liu et al. [24] isolated two *CAT* genes from sugarcane, including *SoCAT-1* in *S. officinarum* and *SsCAT-1* in *S. spontaneum*, and found two allelic variants at the *SoCAT-1* (*SoCAT-1a* and *SoCAT-1b*, GenBank Accession Nos. KF864224 and KF864225) and *SsCAT-1* (*SsCAT-1a* and *SsCAT-1b*, GenBank Accession Nos. KF864226 and KF864227) loci. Liu et al. [25] have also cloned *EaCAT-1b* (GenBank Accession No. KF864228) from *Erianthus arundinaceus* and *SoCAT-1c* (GenBank Accession No. KF864231) from *S. officinarum*. Although the cDNA homology between *EaCAT-1b* and *SoCAT-1c* is 98.6%, *EaCAT-1b* is upregulated by drought stress, whereas *SoCAT-1c* is downregulated. However, information on the full-length coding sequence of other *ScCAT* family members and their functions in sugarcane defense is limited. In the present study, a full-length sugarcane catalase gene *ScCAT2* (GenBank Accession No. KF528830) was isolated from Yacheng05-179. After protein structural prediction and phylogenetic reconstruction, the gene expression patterns of *ScCAT2* under biotic and abiotic stresses, prokaryotic expression analysis, subcellular localization, and transient overexpression in *N. benthamiana* plants treated with tobacco pathogens, were investigated.

## 2. Results

### 2.1. Cloning and Sequence Analysis of ScCAT2 Gene

**Figure 1:**
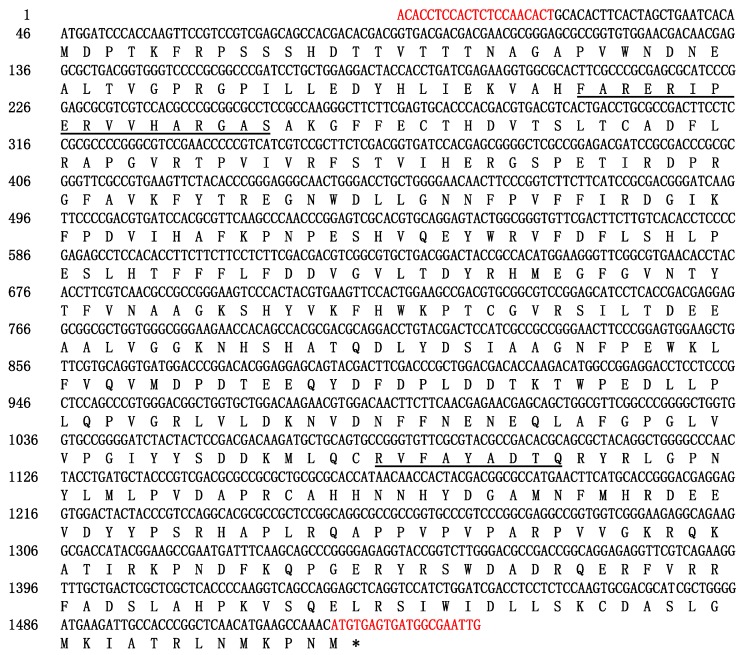
Nucleotide acid sequence and the deduced amino acid sequence of *ScCAT2*. Red letters represented the specific amplification primer pair for *ScCAT2*. Catalase active site (FARERIPERVVHARGAS) and heme-ligand (RVFAYADTQ) signatures of ScCAT2 were underlined. * represented stop codon.

**Figure 2:**
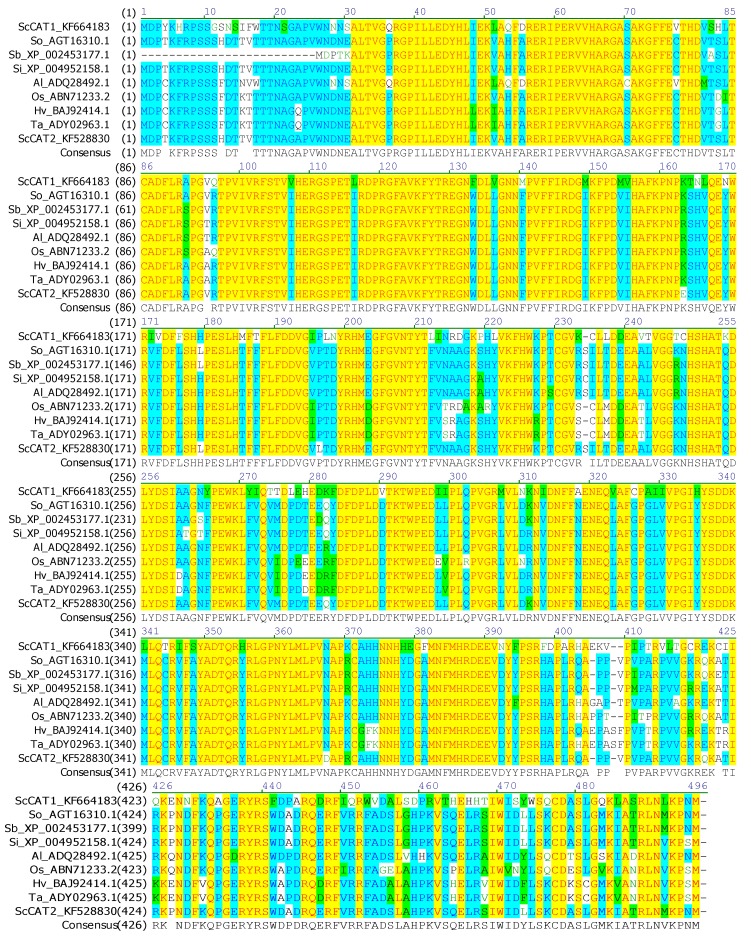
Protein sequence and homology of ScCAT2 with catalases from sugarcane and other plant spices. So, *Saccharum officinarum*; Sb, *Sorghum bicolor*; Si, *Setaria italic*; Al, *Aeluropus litoralis*; Os, *Oryza sativa*; Hv, *Hordeum vulgare*; and Ta, *Triticum aestivum*. The black foreground and white background represented non-similar residues. The blue foreground and cyan background indicated conservative residues. The black foreground and green background were blocks of similar residues. The red foreground and yellow background showed identical residues. The dark green foreground and white background depicted the weakly similar residues.

**Figure 3:**
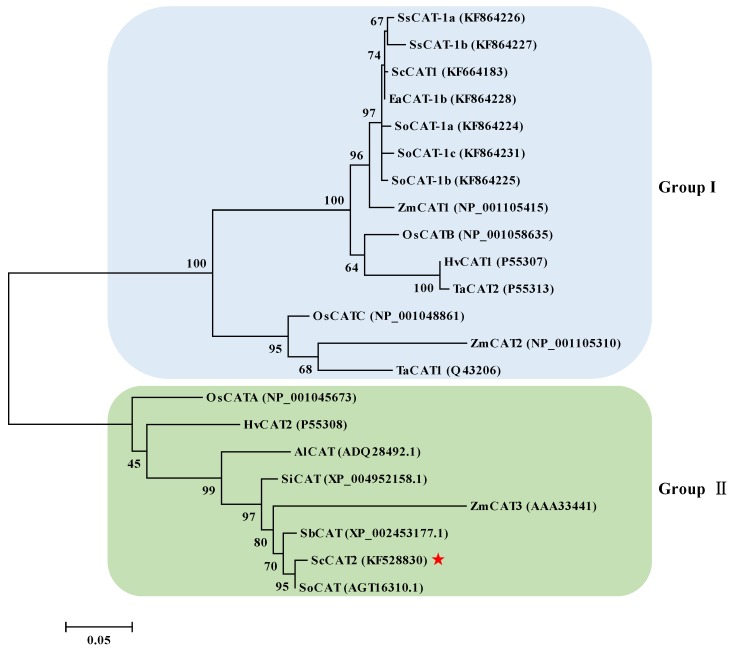
Phylogenetic reconstruction of ScCAT2 with catalases from sugarcane and other plant species. The maximum-likelihood method with 1000 bootstrap replication was used. Zm, *Zea mays*; Os, *Oryza sativa*; Hv, *Hordeum vulgare*; Ta, *Triticum aestivum*; Sb, *Sorghum bicolor*; Si, *Setaria italic*; Al, *Aeluropus littoralis*; So, *Saccharum officinarum*; Ss, *Saccharum spontaneum*; and Ea, *Erianthus arundinaceus*. ScCAT2 was marked with a red asterisk.

A full-length sugarcane catalase gene *ScCAT2* (GenBank Accession No. KF528830) was assembled in silico, and cloned from Yacheng05-179 by real-time polymerase chain reaction (RT-PCR). The open reading frame (ORF) of the *ScCAT2* gene consisted of 1482 nucleotides and was predicted to encode 493 amino acids (Figure 1). The molecular weight and isoelectric point of the ScCAT2 protein were 56.51 kDa and 6.32, respectively. The ScCAT2 protein was hydrophilic and did not include a signal peptide and transmembrane helix domain, indicating that ScCAT2 may not be a secretory protein. The results of conservative domain search result suggested that ScCAT2 was a member of the catalase-like superfamily which was characterized by a catalase active site signature (FARERIPERVVHARGAS) at positions 54–70, and a heme-ligand signature (RVFAYADTQ) at positions of 344–352 (Figure 1). The secondary structure of the protein encoded by the *ScCAT2* gene consisted of irregular curls (57%), α-spirals (25.15%), and extension chains (17.85%). Basic local alignment search tool for proteins (BLASTP) sequence analysis indicated that ScCAT2 was highly homologous to sugarcane and other plant catalases (Figure 2), including the catalase (AGT16310.1, 99.19% identity) from *Saccharum* hybrid cultivar R570, *Sorghum bicolor* catalase (XP_002453177.1, 91.68% identity), *Setaria italic* catalase (XP_004952158.1, 96.35% identity), *Aeluropus littoralis* catalase (ADQ28492.1, 90.89% identity), *O. sativa* catalase (ABN71233.2, 87.63% identity), *H. vulgare* catalase (BAJ92414.1, 87.68% identity), *T. aestivum* catalase (ADY02963.1, 87.88% identity), and *S. officinarum* ScCAT1 (KF664183, 71.81% identity). Figure 3 showed that the catalase proteins from sugarcane, *Z. mays*, *O. sativa*, *H. vulgare*, *T. aestivum*, *S. italic*, *S. bicolor*, and *A. litoralis* could be clustered into two groups, which agreed with the results of Saruyama and Matsumura [6]. ScCAT2 was clustered with group II, and only showed 71.81% identity with ScCAT1, which belonged to group I. These results suggest that sugarcane ScCAT2 encodes a hypothetical group II catalase.

### 2.2. Subcellular Localization of ScCAT2

**Figure 4:**
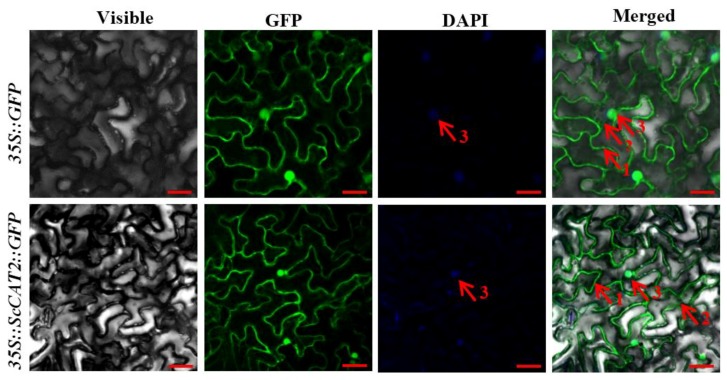
Subcellular localization of ScCAT2 and empty vector in *Nicotiana benthamiana* leaves after 2 days of infiltration. Images of epidermal cells captured using visible light, green fluorescence, blue fluorescence, and merged light. Red arrows 1, 2, and 3, indicated the plasma membrane, cytoplasm, and nucleus, respectively. Bar = 20 μm. DAPI (4′,6-diamidino-2-phenylindole) was used to stain the nucleus.

*ScCAT2* was constructed on a plant expression vector pCAMBIA 2300 between the *35S* promoter and green fluorescent protein reporter gene (*GFP*), and confirmed by enzyme digestion (Figure S1). After agroinfiltration for 2 days, the GFP of the control and the ScCAT2::GFP fusion protein were localized to the cytoplasm, cell membrane, and nucleus (Figure 4). These results are roughly similar to the protein subcellular localization prediction tool (PSORT) prediction that the ScCAT2 protein was situated in the cytoplasm, plasma membrane, and nucleus, with probabilities of 73.9%, 17.4%, and 4.3%, respectively.

### 2.3. Tissue-Specific Expression Analysis of ScCAT2

**Figure 5:**
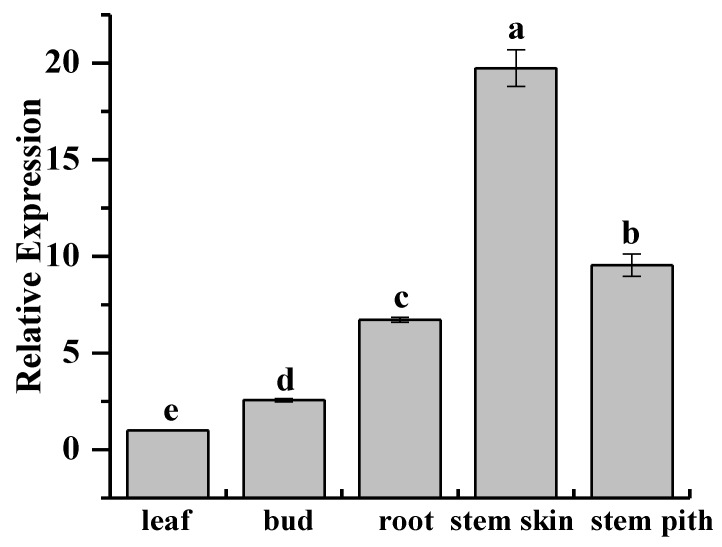
Tissue-specific expression analysis of *ScCAT2* in 10-month-old sugarcane Yacheng05-179 plants. Data were normalized to the expression level of the glyceraldehyde-3-phosphate dehydrogenase (*GAPDH*) gene. All data points were expressed as means ± standard error (*n* = 3). Different lowercase letters indicated a significant difference (*p* < 0.05) compared to the control, as determined with Duncan’s test.

Quantitative real-time polymerase chain reaction (qRT-PCR) analysis showed that *ScCAT2* was ubiquitously expressed in the stem skin, stem pith, roots, buds, and leaves of 10-month-old sugarcane genotype Yacheng05-179 tissues at variable levels (Figure 5). The highest relative *ScCAT2* expression occurs in the stem skin, which was 19.70-fold higher than that in the leaves.

### 2.4. Analysis of the ScCAT2 Expression Patterns in Different Stress Conditions

**Figure 6:**
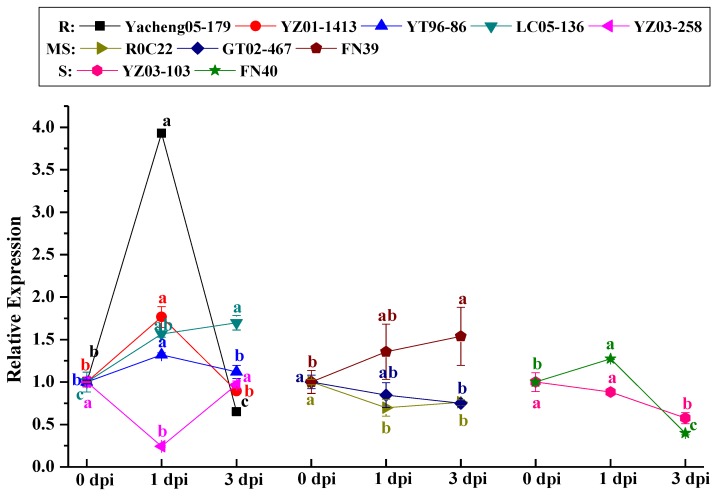
qRT-PCR analysis of the *ScCAT2* expression in 10 different sugarcane genotypes after inoculation with *Sporisorium scitamineum*. The data were normalized to the expression level of the glyceraldehyde-3-phosphate dehydrogenase (*GAPDH*) gene. All data points (normalized to the control) were expressed as means ± standard error (*n* = 3). Different lowercase letters indicated a significant difference (*p* < 0.05) compared to the control, as determined with Duncan’s test. Yacheng05-179, YZ01-1413, YT96-86, LC05-136, and YZ03-258 were smut-resistant cultivars (R). ROC22, GT02-467, and FN39 were smut-medium-susceptibility cultivars (MS). YZ03-103 and FN40 were smut-susceptible cultivars (S). dpi, days post-inoculation.

Figure 6 showed *ScCAT2* transcript abundance in 10 different sugarcane cultivars post-inoculation with *S. scitamineum* as detected by qRT-PCR. Among the five smut-resistant cultivars, compared to the control, YZ03-258 showed a 0.24-fold decrease in *ScCAT2* in expression as early as 1 day post-inoculation (dpi), whereas those of Yacheng05-179, YZ01-1413, YT96-86, and LC05-136 significantly increased by 3.93-, 1.77-, 1.32-, and 1.56-fold. At 3 dpi, the *ScCAT2* transcripts remained at a high level (1.68-fold) in LC05-136, and reverted to the control level in YZ01-1413, YT96-86, and YZ03-258, whereas it decreased by 0.65-fold in Yacheng05-179. In three smut-medium-susceptibility cultivars (ROC22, GT02-467, and FN39) and the two smut-susceptible cultivars (YZ03-103 and FN40), except for the upregulation of FN40 at 1 dpi (1.27-fold) and FN39 at 1–3 dpi (1.36-, and 1.54-fold), *ScCAT2* was downregulated or remained at the control level.

**Figure 7:**
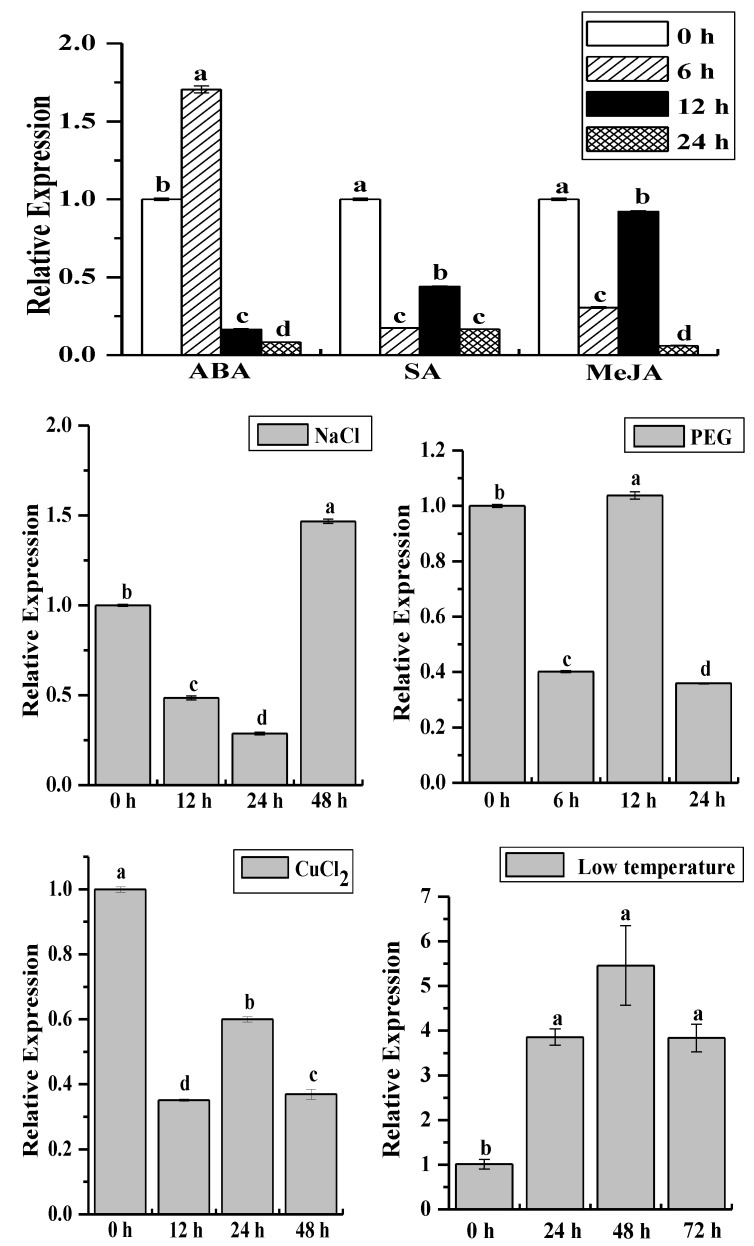
Relative expression of *ScCAT2* after the application of exogenous plant hormone and abiotic stress. *ScCAT2* transcript abundance in Yacheng05-179 tissue cultured plantlets was assessed in the presence of 100 μM ABA, 5 mM SA, 25 μM MeJA, 250 mM NaCl, 25% PEG, 100 μM CuCl_2_, and 4 °C low temperature. The data were normalized to the expression level of the glyceraldehyde-3-phosphate dehydrogenase (*GAPDH*) gene. All data points were expressed as the means ± standard error (*n* = 3). Different lowercase letters indicated a significant difference (*p* < 0.05) compared to the control, as determined with Duncan’s test. ABA, abscisic acid; SA, salicylic acid; MeJA, methyl jasmonate; NaCl, sodium chloride; PEG, polyethylene glycol; CuCl_2_, copper chloride; and low temperature, 4 °C cold stress.

Under exogenous plant hormone and abiotic stress (Figure 7), *ScCAT2* expression was downregulated after treatment with 5 mM SA, 25 μM methyl jasmonate (MeJA), and 100 μM copper chloride (CuCl_2_). After 100 μM ABA treatment, *ScCAT2* transcript abundance significantly increased, as 1.71-fold higher than the control at 6 h, and then underwent a 0.16- and 0.08-fold decrease from 12 h to 24 h. For 250 mM sodium chloride (NaCl) treatment, compared to the control, *ScCAT2* expression was decreased by 0.48- and 0.29-fold at 12–24 h, then increased by 1.47-fold at 48 h. The application of 25% polyethylene glycol (PEG) resulted in a downregulation of *ScCAT2* at 6 and 24 h, whereas the highest expression was observed at 12 h, which was 1.04-fold higher than the control. After cold (4 °C low temperature) treatment, *ScCAT2* transcript abundance significantly increased at 24 h and then kept at a certain level from 24 to 72 h, which was 3.82-, 5.40-, and 3.79-fold higher than the control. Taken together, *ScCAT2* expression in sugarcane decreases or does not change with SA, MeJA, and CuCl_2_ application, but increases with ABA, NaCl, PEG, and cold stimuli.

### 2.5. Prokaryotic Expression Analysis of ScCAT2

**Figure 8:**
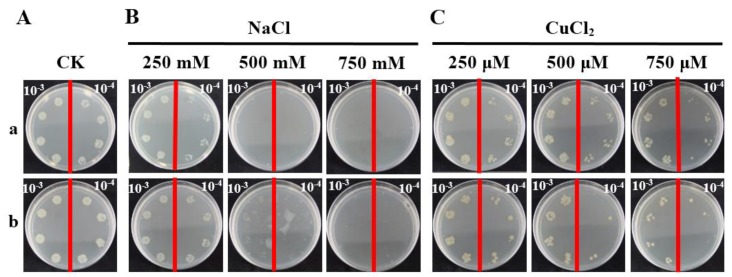
Spot assay of Rosetta + pET-32a (control) (**a**) and Rosetta + pET-32a-*ScCAT2* (**b**) on LB plates with different concentrations of NaCl and CuCl_2_ components. The cultures of Rosetta + pET-32a-*ScCAT2* and Rosetta + pET-32a were supplemented with 1.0 mM isopropyl β-d-thiogalactoside to induce the expression of recombinant protein overnight. Then, the cultures were adjusted to an OD_600_ = 0.6. Ten microliters from the 10^−3^ (left side of red line on plate) to 10^−4^ (right side of red line on plate) dilutions were spotted onto the LB basal plates (**A**) or with NaCl (250, 500, and 750 mM) (**B**), and CuCl_2_ (250, 500, and 750 μM) (**C**). NaCl, sodium chloride; and CuCl_2_, copper chloride.

To investigate the response of the *ScCAT2* gene to adverse environmental factors, pET-32a-*ScCAT2* (Figure S2A) was transformed into *E. coli* Rosetta cells. Sodium dodecyl sulfate polyacrylamide gel electrophoresis (SDS-PAGE) analysis identified a recombinant protein with a molecular mass of >60 kDa after 2 h post-isopropyl β-d-thiogalactoside (IPTG) induction (Figure S2B). After culturing overnight, the colonies of Rosetta + pET-32a-*ScCAT2* that were cultured on CuCl_2_-supplemented LB plates were nearly the same as that of the control. Interestingly, a higher number of Rosetta + pET-32a-*ScCAT2* colonies were detected than the control LB plates containing NaCl (Figure 8). This result indicates that the ScCAT2 recombinant protein enhances the growth rate of the prokaryotic *E. coli* Rosetta strain under NaCl stress, but not under CuCl_2_ stress.

### 2.6. Transient Expression of ScCAT2 in N. benthamiana-Induced Plant Immune Responses

**Figure 9:**
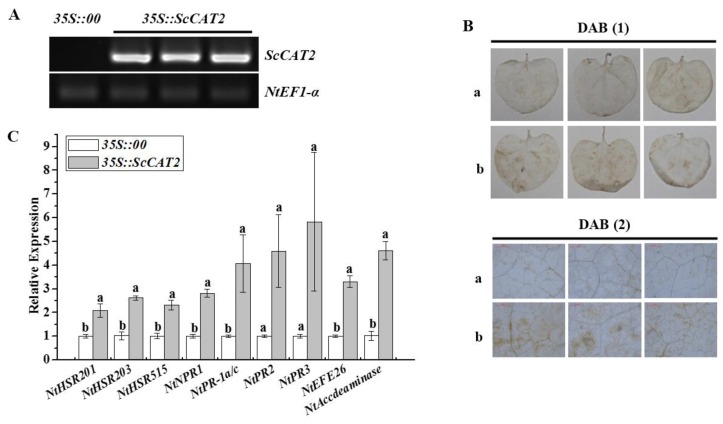
Transient overexpression of *ScCAT2* in *Nicotiana benthamiana* leaves. (**A**) RT-PCR analysis of *ScCAT2* in the *N. benthamiana* leaves 2 days after infiltration with *Agrobacterium* strain GV3101 that carried the vector *35S::00* or *35S::ScCAT2*. (**B**) DAB staining of *N. benthamiana* leaves 2 days after infiltration with *35S::ScCAT2*-containing *Agrobacterium* strain to assess H_2_O_2_ production. (**C**) Relative expression level of nine tobacco immune-related marker genes in *35S::ScCAT2*-transiently expressing leaves at 2 days after infiltration. The tobacco immune-related marker genes included the hypersensitive response marker genes *NtHSR201*, *NtHSR203*, and *NtHSR515*; the salicylic acid-related genes *NtPR-1* and *NtPR-1a/c*; the jasmonic acid pathway-associated genes *NtPR2* and *NtPR3*; and the ethylene synthesis-dependent genes *NtEFE26* and *NtAccdeaminase*, and using *NtEF1-α* for data normalization. All data points were presented as the means ± standard error (*n* = 3). Different lowercase letters indicated a significant difference, as determined with Duncan’s test (*p* < 0.05). The empty vector *35S::00* and recombinant vector *35S::ScCAT2* were indicated by a and b, respectively. (1) and (2) represented images captured using a SONY camera and microscope, respectively. Bar = 1 mm.

The empty vector pCAMBIA 1301 and the recombinant vector pCAMBIA 1301-*ScCAT2* (Figure S3) were transformed into *Agrobacterium* Gv3101 and injected into *N. benthamiana* leaves. Figure 9A showed that at 2 days post-agroinfiltration (dpai), *ScCAT2* transcript abundance increased, as indicated by RT-PCR analysis. A typical allergic reaction, as depicted by a darker 3,3′-diaminobenzidine (DAB) staining color, was observed on the leaves, in which *ScCAT2* was transiently overexpressed (Figure 9B). In addition, qRT-PCR was used to analyze the expression of nine tobacco immune-related marker genes in *N. benthamiana* leaves at 2 dpai. The results showed that the HR marker genes *NtHSR201*, *NtHSR203*, and *NtHSR515*; the SA-related genes *NtPR1* and *NtPR-1a/c*; the JA pathway-associated genes *NtPR2* and *NtPR3*; and the ethylene (ET) synthesis-dependent genes *NtEFE26* and *NtAccdeaminase*, were all upregulated, which were 2.07-, 2.56-, 2.28-, 2.81-, 4.07-, 4.58-, 5.80-, 3.30-, and 4.51-fold higher than the control, respectively (Figure 9C). These results indicate that the transient overexpression of *ScCAT2* in the *N. benthamiana* leaves induces plant allergic reactions.

**Figure 10:**
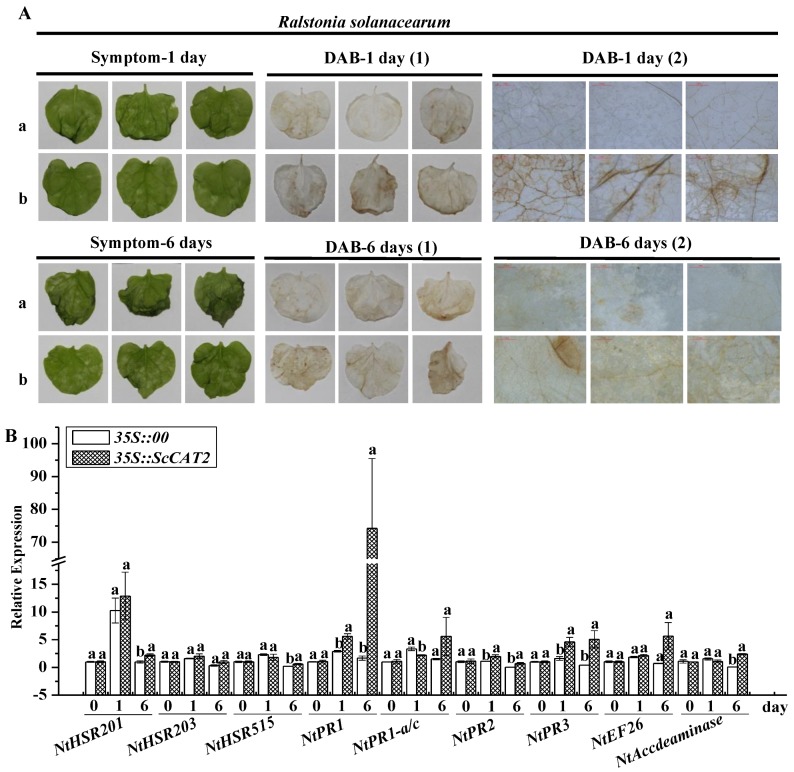
The effect of transient overexpression of *ScCAT2* in *Nicotiana benthamiana* leaves after inoculation with *Ralstonia solanacearum*. (**A**) The disease symptoms and DAB staining of *N. benthamiana* leaves at 1 day and 6 days post-inoculation with *R. solanacearum*. (**B**) Analysis of nine tobacco immune-related marker genes in the *N. benthamiana* leaves 1 day and 6 days after inoculation with *R. solanacearum*. The tobacco immune-related marker genes, which included the hypersensitive response marker genes *NtHSR201*, *NtHSR203*, and *NtHSR515*; the salicylic acid-related genes *NtPR-1* and *NtPR-1a/c*; the jasmonic acid pathway-associated genes *NtPR2* and *NtPR3*; and the ethylene synthesis-dependent genes *NtEFE26* and *NtAccdeaminase*, and using *NtEF1-α* for data normalization. All data points were presented as means ± standard error (*n* = 3). Different lowercase letters indicated a significant difference, as determined with Duncan’s test (*p* < 0.05). The empty vector *35S::00* and recombinant vector *35S::ScCAT2* were indicated by a and b, respectively. (1) and (2) represented images captured using a SONY camera and microscope, respectively. Bar = 1 mm.

To test the response of the *ScCAT2* gene to plant pathogens, two major tobacco pathogens, namely, *Ralstonia solanacearum* and *Fusarium solani* var. *coeruleum*, were separately inoculated into *N. benthamiana* leaves, which were infiltrated with the *Agrobacterium* GV3101 strain that carries *35S::00* or *35S::ScCAT2* for 1 day. After challenging with *R. solanacearum* for 1 day, weak symptoms of disease were observed in the control leaves as indicated by the intensity of DAB staining, whereas in the *35S::ScCAT2* leaves, a higher-intensity of DAB staining, indicative of a more severe level of disease, was observed (Figure 10A). At 6 dpai, the control leaves exhibited more severe necrotic spots, whereas the *35S::ScCAT2* leaves only showed minimal wilting. Additionally, the *35S::ScCAT2* leaves exhibited more intense DAB staining than the control. Moreover, compared to the control, the *35S::ScCAT2* leaves showed upregulated *NtPR1*, *NtPR2*, and *NtPR3* levels both at 1 and 6 dpai. No changes in *NtHSR201*, *NtHSR515*, and *NtAccdeaminase* transcript abundance were observed at 1 dpai, but these increased at 6 dpai. The expression levels of *NtHSR203* and *NtEFE26* did not change at 1 and 6 dpai, and *NtPR-1a/c* was downregulated at 1 dpai (Figure 10B).

**Figure 11:**
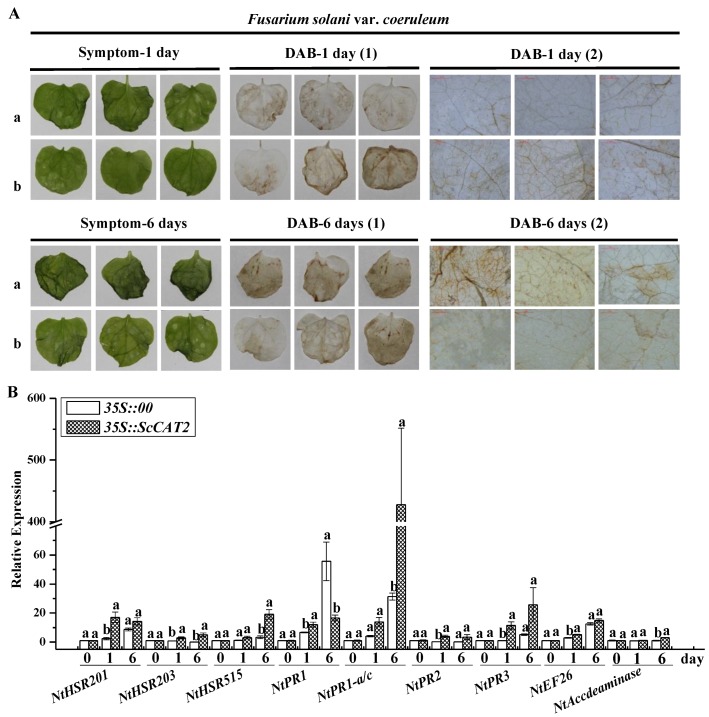
The effect of transient overexpression of *ScCAT2* in *Nicotiana benthamiana* leaves after inoculation with *Fusarium solani* var. *coeruleum*. (**A**) The disease symptoms and DAB staining of *N. benthamiana* leaves 1 day and 6 days after inoculation with *F. solani* var. *coeruleum*. (**B**) Analysis of the nine tobacco immune-related marker genes in the *N. benthamiana* leaves after inoculation with *F. solani* var. *coeruleum* for 1 day and 6 days, respectively. The tobacco immune-related marker genes included the hypersensitive response marker genes *NtHSR201*, *NtHSR203*, and *NtHSR515*; the salicylic acid-related genes *NtPR-1* and *NtPR-1a/c*; the jasmonic acid pathway-associated genes *NtPR2* and *NtPR3*; and the ethylene synthesis-dependent genes *NtEFE26* and *NtAccdeaminase*, and using *NtEF1-α* for data normalization. All data points were means ± standard error (*n* = 3). Different lowercase letters indicated a significant difference, as determined with Duncan’s test (*p* < 0.05). The empty vector *35S::00* and recombinant vector *35S::ScCAT2* were indicated by a and b, respectively. (1) and (2) represented images captured using a SONY camera and microscope, respectively. Bar = 1 mm.

Similarly, after injecting with *F. solani* var. *coeruleum* and compared to the *35S::ScCAT2* leaves, the control leaves exhibited more severe disease symptom at 1 and 6 dpai. In terms of DAB staining, the *35S::ScCAT2* leaves were deeper brown at 1 dpai, and lighter brown at 6 dpai (Figure 11A). Figure 11B showed that compared to the control, *NtHSR201*, *NtPR2*, *NtPR3*, and *NtEFE26* transcript abundance in the *35S::ScCAT2* leaves increased at 1 dpai, and showed the same level as that of the control at 6 dpai. *NtHSR515*, *NtPR-1a/c*, and *NtAccdeaminase* transcript expression in the *35S::ScCAT2* leaves did not change at 1 dpai, but increased at 6 dpai. *NtHSR203* in the *35S::ScCAT2* leaves was upregulated at both 1 and 6 dpai. *NtPR-1* was upregulated in the *35S::ScCAT2* leaves at 1 dpai and downregulated at 6 dpai.

## 3. Discussion

As a multigene family, CATs have been extensively studied for their participation in plant defense, growth, and development [9,11,13,15,16,17,23]. In this study, a full-length *ScCAT2* gene, which encoded a polypeptide consisting of 493 amino acids, was obtained from sugarcane. At the amino acid level, the homology of ScCAT2 to previously reported sugarcane CATs, such as ScCAT1 (KF664183), SoCAT-1a (KF864224), SoCAT-1b (KF864225), SoCAT-1c (KF864231), SsCAT-1a (KF864226), SsCAT-1b (KF864227), and EaCAT-1b (KF864228), were 71.81%, 71.81%, 71.81%, 71.20%, 71.60%, 71.20%, and 71.60%, respectively. The genetic background of sugarcane is complex, as *Saccharum* hybrids are highly polyploid and derived from interspecific hybridization between *S. officinarum* and *S. spontaneum*, and each gene has 8–10 copies [26]. Furthermore, the catalase family also consists of different members that are homologous in sequence. In this study, amino acid sequence homology between ScCAT2 and the catalase (AGT16310.1) from *Saccharum* hybrid cultivar R570 (unpublished) was 99.19%, suggesting that these two genes probably represent two variants. Plant CATs in plants are mainly distributed in the cytoplasm, peroxisomes, and glyoxysomes, and trace amounts have been reported in the mitochondria of plant cells [7,8]. A previous study localized the sugarcane ScCAT1 protein to the plasma membrane and cytoplasm [23]. Iwamoto and Higo [27] predicted that the sense and antisense large *CatB* transcripts in rice might be distributed in the nucleus. In this study, using *Agrobacterium*-mediated transformation, ScCAT2::GFP was localized to the cytoplasm, plasma membrane, and nucleus (Figure 4). Phylogenetic reconstruction indicated that the ScCAT2 protein belonged to group II, and was highly homologous to OsCATA, HvCAT2, AlCAT, SiCAT, ZmCAT3, SbCAT, and SoCAT (Figure 3). The *AlCAT* gene in group II has been reported to play a positive role in salinity responses under NaCl treatment [28]. Normal *ZmCAT3* transcript levels were observed in both pigment-deficient mutants and the wild-type, whereas the highest expression levels were observed in the leaves, suggesting that *ZmCAT3* is involved in non-photosynthetic reactions [11,29]. Furthermore, Guan and Scandalios [30] reported that *ZmCAT3* is downregulated in leaves after the application of ABA and osmotic stress.

Previous studies have shown that catalases exhibit tissue-specific differential expression in plants [31,32,33]. Higher *NnCAT* expression levels have been reported in young leaves of *Nelumbo nucifera* compared to other tissues, such as the roots, terminal buds, and leafstalks [31]. In *A. thaliana*, the expression pattern of *CAT1*, *CAT2*, and *CAT3* was tissue-specific and age-dependent, unlike *CAT2* and *CAT3*, and *CAT1* was also expressed in older leaves, flowers, and seeds [33]. *HuCAT3* was constitutively expressed in the mature stems, cotyledons, flowers, caulicles, roots, and fruits of *Hylocereus undatus* [32]. Similarly, in our study, *ScCAT2* was ubiquitously expressed and showed the highest expression level in the stem skin (Figure 5), which differs from *ScCAT1* as it has the highest expression level in the buds [23].

*ScCAT1* positively responds to exogenous stimuli, including plant hormones stresses, such as SA, MeJA, and ABA and H_2_O_2_ oxidative stress, PEG and NaCl hyperosmotic stress, and CuCl_2_ abiotic elicitor or metal stress [23]. The recombinant protein expressed by *ScCAT1* in *E. coli* Rosetta cells enhanced the tolerance of host cells to NaCl, CuCl_2_, and CdCl_2_ stresses [23]. *ScCAT2* expression was downregulated under SA, MeJA, and CuCl_2_ stresses, but upregulated under ABA, NaCl, PEG and cold stimuli (Figure 7). Moreover, the Rosetta cells that expressed the *ScCAT2* recombinant protein showed a higher growth rate under NaCl stress than that of the control, but not under CuCl_2_ treatment (Figure 8). ABA is a stress signal that regulates various stress-responsive genes during osmotic imbalance [34]. This finding illustrates that *ScCAT2* may be involved in the response mechanism of salt and drought resistance in sugarcane, which depends on the ABA signal pathway. Similarly, *CsCAT3* in *Cucumis sativus* was induced by different kinds of stresses, such as heat, cold, NaCl, PEG, H_2_O_2_, and ABA treatments, and its overexpression in *E. coli* could increase the tolerance to heat, cold, NaCl, and sorbitol conditions [35]. *MaCat2*, a catalase gene from banana (*Musa acuminate*) fruit, was upregulated by low temperature and physical damage [36]. Nie et al. [32] found that *HuCAT3* was upregulated 7 days after application of drought stress, approximately eight-fold higher than that of the control, and reached a peak value after 6 h exposure to NaCl stress. ABA and osmotic stress increased *ZmCAT1* expression in embryos and leaves [30]. Du et al. [9] reported that three *CATs* gene in *A. thaliana* have different functions under drought, cold, and oxidative stresses, as well as with ABA and SA treatments. *AtCAT1* played an important role in all stresses, *AtCAT2* was upregulated by drought and cold, and *AtCAT3* was mainly upregulated by oxidative and ABA treatments. Three cDNA sequences of the *CAT* genes of *O. sativa*, *OsCatA*, *OsCatB*, and *OsCatC*, were cloned and exhibited variable activity in the presence of different salt stresses (i.e., NaCl, potassium chloride, lithium chloride, and magnesium chloride) at different concentrations (0–1000 mM), with *OsCatC* showing higher tolerance than *OsCatA* and *OsCatB* (<60 mM), whereas the opposite effects were observed when salt concentrations were >125 mM [37]. The differential expression of *ScCAT2* and *ScCAT1* in response to various stresses suggests that *ScCAT2* and *ScCAT1* may coordinately regulate plant development.

*CAT* genes can be regulated by abiotic stress. Ma et al. [38] found that the *LsCat1* gene in *Lilium sargentiae* Wilson was induced after infection with *Fusarium oxysporum*. The expression level of *CAT1* in *Nicotiana tabacum* L. was enhanced by TMV infection [39]. During sugarcane–*S. scitamineum* interaction, *ScCAT1* transcript abundance in the incompatible interaction (Yacheng05-179-*S. scitamineum* interaction) was higher and expressed earlier than the compatible one (Liucheng03-182-*S. scitamineum* interaction) [23]. Here, we examined *ScCAT2* expression in 10 different sugarcane genotypes post-smut pathogen inoculation (Figure 6) and found that, except for YZ03-258, *ScCAT2* transcript abundance in the other four smut-resistant cultivars significantly increased compared to the control, and peaked as early as 1 dpi. However, in the three smut-medium-susceptibility cultivars and two smut-susceptible cultivars, except for the upregulation in FN40 at 1 dpi as well as in FN39 at 1–3 dpi, *ScCAT2* transcript abundance significantly decreased or did not change. Taken together, the results of this study indicate that *ScCAT2* is a positive responsive component of smut resistance in sugarcane, and that *ScCAT2* and *ScCAT1* may act synergistically against *S. scitamineum*.

Increased resistance to *Peronospora parasitica* and *Erysiphe polygoni* has been reported in transgenic canola (*Brassica napus* L.), which overexpressed bacterial catalase [40]. Yu et al. [41] indicated that overexpression of the tobacco *Cat*2 gene in *Solanum tuberosum* increases its tolerance to *Phytophthora infestans*. Transgenic tobacco overexpressing the maize *Cat2* gene showed higher catalase activity, which was relative to plant–pathogen interactions [42]. Previous studies have demonstrated that cell death is a resistance mechanism of plants against infections, and involves a series of cellular and molecular processes, including induction of HR gene expression, increase in reactive oxygen species (ROS) production, accumulation of defense-related hormones, and increase in ion fluxes [43,44,45]. The accumulation of H_2_O_2_ has, thus, been used as indicator for local allergic reactions in plant disease [46]. DAB can react with peroxide to produce a bronze precipitate, and the intensity of staining in the leaves reflects the degree of H_2_O_2_ accumulation [43]. The gradation of DAB staining has also been employed as an index for cell death during hypersensitivity responses [43]. *Agrobacterium*-mediated stable transformation, including gene overexpression and RNAi, is a common method to study the functions of target genes. However, the transformation efficiency and regeneration efficiency are too low and time-consuming to characterize multiple genes. In recent years, the techniques of gene silencing and transient expression based on agroinfiltration have become important methods for gene function research, because of their short time consumption and high throughput [47,48,49]. In previous study, *ScCAT1* has been shown to play a positive role in HR and immune responses by transient overexpression in *N. benthamiana* leaves [23]. Similarly, in our study, the control leaves post-inoculated with *R. solanacearum* or *F. solani* var. *coeruleum* exhibited more severe disease symptoms than the leaves that overexpressed *ScCAT2* (Figure 10 and Figure 11). Furthermore, these showed more intense DAB staining, and transcript abundance of most of the tested tobacco immune-related marker genes was higher in the *35S::ScCAT2* leaves than the control (Figure 9, Figure 10 and Figure 11). These results reveal that the *ScCAT2* gene plays a positive role in plant defense responses, and the overexpression of *ScCAT2* in *N. benthamiana* leaves enhances resistance to *R. solanacearum* and *F. solani* var. *coeruleum* infections.

As is known, *Saccharum* spp., are members of a complex genus characterized by high polyploidy, frequent aneuploidy and heterozygosity, large genomes, low fertility rates, and long growth periods [26,50,51]. These factors make it a prime candidate for improvement through genetic engineering instead of traditional breeding, but at the same time, may impact the expression and heredity stabilization of target genes in transgenic sugarcane, and result in sugarcane transgenic technology with low transformation efficiency and long experimental periods [52,53,54]. Thus, whether *ScCAT2* gene plays a direct causal role in defense against pathogen and/or salt, drought, and cold stimuli needs further transgenic sugarcane investigation, which can be referred to in the methods of Shen et al. [55] and Kumar et al. [56].

## 4. Materials and Methods

### 4.1. Plant Materials and Treatment Conditions

In the sugarcane–*S. scitamineum* biosystem, 10 sugarcane varieties, including five smut-resistant (Yacheng05-179, YZ01-1413, YT96-86, LC05-136, and YZ03-258), three smut-medium-susceptibility (ROC22, GT02-467, and FN39), and two smut-susceptible (YZ03-103 and FN40) varieties, were collected from the Key Laboratory of Sugarcane Biology and Genetic Breeding, Ministry of Agriculture (Fuzhou, China) [57]. Smut whips were harvested in the most popular cultivar ROC22 in our laboratory, and stored at 4 °C. Physically similar sugarcane stems from the 10 sugarcane varieties were collected and cut into two-bud setts. After immersing in flowing water for 1 day, the sugarcane segments were cultivated in an incubator under a light–dark regime (16 h of light and 8 h of darkness) at 32 °C. When the germinating seedlings developed buds with a height of about 2 cm, these were inoculated by acupuncture with a suspension of 5 × 10^6^ spores/mL containing 0.01% (*v*/*v*) Tween-20, whereas controls were mock-inoculated with sterile distilled water containing 0.01% (*v*/*v*) Tween-20 [58,59]. All inoculated materials were grown at 28 °C with a photoperiod of 16 h light and 8 h darkness. Five buds at time points of 0, 1, 3, and 7 dpi were harvested and immediately frozen in liquid nitrogen, and stored at −80 °C until use.

Six consistent 10-month-old sugarcane Yacheng05-179 plants showing similar growth rates were randomly selected from the fields and used in tissue-specific expression analysis. The youngest fully expanded leaf (+1 leaf) with a visible dewlap (the collar between the leaf blade and sheath), buds, white roots, stem skin, and stem pith from the sugarcane seventh or eighth nodes were collected [60]. All samples were frozen in liquid nitrogen, and then stored in a −80 °C refrigerator until RNA extraction.

Four-month-old Yacheng05-179 tissue-cultured plantlets were transferred to water for one week. The six following chemical stimuli were applied and kept at 28 °C with a photoperiod of 16 h light and 8 h darkness. The leaves of three groups of Yacheng05-179 plantlets were sprayed with 100 μM ABA, 5 mM SA (containing 0.01% Tween-20, *v*/*v*), and 25 μM MeJA (containing 0.1% ethanol and 0.05% Tween-20, *v*/*v*) [23]. The other three groups were grown hydroponically in a solution containing 250 mM NaCl, 25% PEG 8000, and 100 μM CuCl_2_ [23]. Whole plantlets were harvested at 0, 6, 12, and 24 h after treatment with ABA, SA, MeJA, and PEG. The other plantlets treated with NaCl or CuCl_2_ were collected at 0, 12, 24, and 48 h. For cold stress, the whole Yacheng05-179 plantlets were kept at a low temperature of 4 °C, with a photoperiod of 16 h light and 8 h darkness for 0, 24, 48, and 72 h. The samples were immediately frozen in liquid nitrogen and then stored at −80 °C until RNA extraction. All treatments were conducted using three biological replicates.

### 4.2. Total RNA Extraction and Synthesis of the First-Strand cDNA

Total RNA was extracted from the collected tissues using TRIzol™ (Invitrogen, Carlsbad, CA, USA). DNA contamination was removed using DNase I (Promega, Beijing, China). The Prime-Script™ RT Reagent Kit (TaKaRa, Dalian, China) was used to reverse transcribe RNA (1.0 μg) into first-strand cDNA.

### 4.3. Cloning of ScCAT2 Gene

A putative cDNA sequence of the *ScCAT2* gene was assembled, in silico, using a probe sequence of the sugarcane *CAT* gene expressed sequence tag (GenBank Accession No. CF573140.1) in NCBI [23]. The *ScCAT2* gene was amplified using the primer pair *ScCAT2*-cDNAF/*ScCAT2*-cDNAR (Table S1), based on the bud cDNA template of Yacheng05-179 in a Mastercycler (Eppendorf, Hamburg, Germany). The RT-PCR temperature conditions were 94 °C for 5 min, followed by 35 cycles of 94 °C for 30 s, 55 °C for 45 s, and 72 °C for 2 min, and an elongation step at 72 °C for 10 min. The PCR products were gel-purified, cloned into pMD18-T vector (TaKaRa, Dalian, China) and sequenced (Shenggong, Shanghai, China).

### 4.4. Protein Structural Analysis and Phylogenetic Reconstruction

The ORF of the *ScCAT2* gene was analyzed using ORF Finder (available online: https://www.ncbi.nlm.nih.gov/orffinder/). ProtParam (available online: http://web.expasy.org/protparam), GOR IV (available online: https://npsa-prabi.ibcp.fr/cgi-bin/npsa_automat.pl?page=/NPSA/npsa_hnn.html), SignalP 4.0 Server (available online: http://www.cbs.dtu.dk/services/SignalP), ProtFun (available online: http://www.cbs.dtu.dk/services/ProtFun/), TMHMM Server v. 2.0 (available online: http://www.cbs.dtu.dk/services/TMHMM/), PSORT (available online: http://www.genscript.com/psort.html), ProtScale (available online: http://web.expasy.org/protscale/), and MOTIF Search (available online: http://www.genome.jp/tools/motif/) programs were used to predict the physical and chemical parameters, protein secondary structure, the signal peptide sequences and the precise location of the cleavage sites, the cellular role and enzyme class and Gene Ontology category, the transmembrane helices, protein subcellular localization, and the motifs in the amino acid sequences of ScCAT2. After BLAST analysis, the DNAMAN and NTI software were used to conduct multiple alignment of the deduced amino acid sequence of the *ScCAT2* gene with the other catalases, including *ScCAT1* (KF664183) from *Saccharum* hybrid cultivar Yacheng05-179, catalase (AGT16310.1) from *Saccharum* hybrid cultivar R570, *S. bicolor* catalase (XP_002453177.1), *S. italic* catalase (XP_004952158.1), *Aeluropus litoralis* catalase (ADQ28492.1), *O. sativa* catalase (ABN71233.2), *H. vulgare* catalase (BAJ92414.1), and *T. aestivum* catalase (ADY02963.1). The MEGA 6 software was used to construct a phylogenetic tree using the neighbor-joining (NJ) method (1000 bootstrap replicates) [61].

### 4.5. Subcellular Localization Assay

The plasmid PMD18-T-*ScCAT2* was used as template to amplify the ORF fragment of *ScCAT2* without a stop codon, using primers *ScCAT2*-SublocF/*ScCAT2*-SublocR (Table S1). Thereafter, *ScCAT2* was inserted into the plant expression vector pCAMBIA 2300-*GFP* via restriction endonuclease (*Xba*I and *Spe*I) and T4 DNA ligase. The positive plasmid pCAMBIA 2300-*ScCAT2*-*GFP* was confirmed by enzyme digestion, and transformed into *Agrobacterium tumefaciens* EHA105 strain. The EHA105 cells carrying pCAMBIA 2300-*ScCAT2*-*GFP* or pCAMBIA 2300-*GFP* were cultured in liquid LB medium containing 50 μg/mL kanamycin and 35 μg/mL rifampicin. Then, the concentration of the suspension was adjusted to an OD_600_ = 0.8 using MS liquid medium containing 200 μM acetosyringone. The four-to five-week-old *N. benthamiana* leaves were selected for injection. After cultivating at 28 °C for 2 days (16 h light and 8 h darkness), subcellular localization of the GFP reporter protein was determined by confocal laser scanning microscopy using a Leica TCS SP5 (Wetzlar, Germany).

### 4.6. Quantification of ScCAT2 Expression by qRT-PCR Analysis

Changes in *ScCAT2* expression in the 10 sugarcane varieties after inoculation with smut pathogen were determined by qRT-PCR. *ScCAT2* transcript abundance of the inoculated material minus the gene expression level of the control group was determined at various time points to eliminate the influence of mechanical damage. The transcripts of *ScCAT2* in different sugarcane tissues (leaves, buds, roots, stem skin, and stem pith) and under exogenous plant hormones (ABA, SA, and MeJA) and abiotic stresses (NaCl, PEG, and CuCl_2_) were also assessed using qRT-PCR. The qRT-PCR primer pair *ScCAT2*-QF/*ScCAT2*-QR (Table S1) was designed by Primer Premier 5.0. The glyceraldehyde-3-phosphate dehydrogenase (*GAPDH*) gene (Table S1) was used as reference [62,63]. qRT-PCR was performed on an ABI 7500 system using the SYBR-green dye method. The total volume of the qRT-PCR reaction system was 25 μL, which included 12.5 μL of the FastStart Universal SYBR Green PCR Master (ROX), 0.4 μmoL of the primer, and 2.0 μL of the template (10× cDNA diluted liquid). The qRT-PCR conditions were 50 °C for 2 min, and 95 °C for 10 min, followed by 40 cycles of 95 °C for 15 s, and 60 °C for 1 min. Each sample was assessed using three replicates, using sterile water as control. Expression levels were calculated using the 2^−ΔΔ*C*T^ algorithm [64].

### 4.7. Prokaryotic Expression Assay

The ORF fragment of *ScCAT2* was amplified from plasmid PMD18-T-*ScCAT2* using the primer pair *ScCAT2*-32aF/*ScCAT2*-32aR (Table S1). The PCR program was 94 °C for 4 min; followed by 35 cycles of 94 °C for 30 s, 58 °C for 30 s, 72 °C for 90 s; and then, a final steps of 72 °C for 10 min. The PCR product and the prokaryotic expression vector pET-32a (+) were digested with *Eco*RI and *Xho*I and linked by T4 DNA ligase. The recombinant vector pET-32a-*ScCAT2* was confirmed by enzyme digestion and transformed into the *E. coli* Rosetta strain cells. Single colonies were inoculated into LB liquid medium containing 80 μg/mL ampicillin and 170 μg/mL chloramphenicol, and incubated overnight at 37 °C at 200 rpm. The *E. coli* Rosetta and Rosetta + pET-32a were used as blank and negative control, respectively. The next day, the amount of 1.0% bacteria cells were transferred into fresh LB liquid medium containing the corresponding antibiotics and cultivated at 37 °C at 200 rpm until its OD_600_ reached 0.6. The target proteins were induced by 1.0 mM IPTG at 37 °C for 2 h, and then subjected to 12% SDS-PAGE [59].

The Rosetta strains transformed with pET-32a or pET-32a-*ScCAT2* were incubated in LB liquid medium (plus 170 μg/mL chloramphenicol and 80 μg/mL ampicillin). When the OD_600_ of the cells reached 0.6, 1.0 mM IPTG was added and incubated overnight at 28 °C at 200 rpm. Thereafter, the suspension was diluted to an OD_600_ = 0.6, and then to two levels of 10^−3^ and 10^−4^ with fresh LB liquid medium. Ten-microliter aliquots of the 10^−3^ and 10^−4^ dilutions were collected and spotted onto LB plates (plus 170 μg/mL chloramphenicol and 80 μg/mL ampicillin) containing NaCl (250, 500, and 750 mM) or CuCl_2_ (250, 500, and 750 μM). The plates were kept at 37 °C overnight, and photographed using a SONY camera (Tokyo, Japan) [59].

### 4.8. Transient Overexpression of ScCAT2 in N. benthamiana in Response to Pathogen Infection

The plant overexpression vector pCAMBIA 1301-*ScCAT2* was constructed using primer pair *ScCAT2*-1301F/*ScCAT2*-1301R (Table S1), *Xba*I and *Spe*I restriction endonucleases, and T4 DNA ligase. The positive plasmid was transformed into *A. tumefaciens* Gv3101 after PCR detection and enzyme digestion verification. The Gv3101 strains carrying the recombinant plasmid pCAMBIA 1301-*ScCAT2* or the empty vector pCAMBIA 1301 (control) in LB liquid medium (plus 50 μg/mL kanamycin and 35 μg/mL rifampicin) were cultured overnight at 28 °C at 200 rpm, respectively. The cultures were collected by centrifugation and resuspended in fresh MS liquid medium (plus 200 μM acetosyringone) to an OD_600_ = 0.8, and then injected into eight-leaf stage *N. benthamiana* leaves [43]. The agroinfiltrated plants were cultivated at 24 °C for 2 days for DAB staining, and relative expression level analysis of the target gene and nine tobacco immune-related marker genes (Table S1) [59]. All treatments were performed with three replicates.

To determine the inhibitory effect of *ScCAT2* on the pathogens, *N. benthamiana* leaves that were agroinfiltrated with pCAMBIA 1301-*ScCAT2* for 1 day were infected with one of two major tobacco pathogens, namely, *R. solanacearum* and *F. solani* var. *coeruleum* [60]. At 1 and 6 dpai, phenotypic observations, DAB staining, and transcript analysis of the tobacco immune-related marker genes were performed according to Liu et al. [65]. All treatments were performed with three replicates.

## 5. Conclusions

In this study, a full-length sugarcane *CAT* gene *ScCAT2* was cloned and identified. *ScCAT2* was constitutively expressed in sugarcane tissues. The ScCAT2 was localized to the cytoplasm, plasma membrane, and nucleus. The *ScCAT2* transcript levels were upregulated by ABA, NaCl, PEG, and cold stress, but downregulated by SA, MeJA, and CuCl_2_. In the 10 different sugarcane genotypes infected with *Sporisorium scitamineum*, except for YZ03-258, FN40, and FN39, *ScCAT2* transcript abundance in the four smut-resistant cultivars (Yacheng05-179, YZ01-1413, YT96-86, and LC05-136) significantly increased at the early stage (1 dpi), but decreased or did not change in the two smut-medium-susceptibility cultivars (ROC22 and GT02-467) and one smut-susceptible cultivar (YZ03-103), from 0 to 3 dpi. In *E. coli* Rosetta cells, the expression of recombinant protein of ScCAT2 enhanced their tolerance to NaCl stress, whereas the opposite was observed after treatment with CuCl_2_. Moreover, analysis indicated that the overexpression of *ScCAT2* in the *N. benthamiana* leaves enhanced resistance to *R. solanacearum* and *F. solani* var. *coeruleum* infections. These results may be used in mining and functional identification of *ScCAT* family gene*s* in sugarcane.

## Supplementary Materials

Supplementary materials can be found at http://www.mdpi.com/1422-0067/19/9/2686/s1.
