# Supplementary material for: The Role of Sugarcane Catalase Gene ScCAT2 in the Defense Response to Pathogen Challenge and Adversity Stress
Source: Int J Mol Sci. 2018 Sep 10;19(9):2686. doi: 10.3390/ijms19092686 (PMC6163996; doi:10.3390/ijms19092686)
Supplement: Supplementary file 1 [file ijms-19-02686-s001.pdf]

Supplementary Materials: The Role of Sugarcane Catalase Gene *ScCAT2* in the Defense Response to Pathogen Challenge and Adversity Stress

Tingting Sun, Feng Liu, Wenju Wang, Ling Wang, Zhuqing Wang, Jing Li, Youxiong Que, Liping Xu and Yachun Su

Table S1. Primers used in this study.

Primer	Sequence	Strategy
ScCAT2-cDNAF	ACACCTCCACTCTCCAACACT	RT-PCR
ScCAT2-cDNAR	CAATTCGCCATCACTCACAT	RT-PCR
ScCAT2-QF	GGAGTGGAAGCTGTTCGTGC	qRT-PCR
ScCAT2-QR	CGCCGTCGTAGTGGTTGTTA	qRT-PCR
GAPDH-QF	CACGGCCACTGGAAGCA	qRT-PCR
GAPDH-QR	TCCTCAGGGTTCCTGATGCC	qRT-PCR
ScCAT2-SublocF	TGCTCTAGAATGGATCCCACCAAGTTC	Subcellular localization vector construction
ScCAT2-SublocR	GGACTAGTCATGTTTGGCTTCATGTTGAG	Subcellular localization vector construction
ScCAT2-32aF	CGGAATTCATGGATCCCACCAAGTT	Prokaryotic expression vector construction
ScCAT2-32aR	CCCTCGAGTCACATGTTTGGCTTCATG	Prokaryotic expression vector construction
ScCAT2-1301F	TGCTCTAGAATGGATCCCACCAAGTTC	Overexpression vector construction
ScCAT2-1301R	GGACTAGTTCACATGTTTGGCTTCATGTT	Overexpression vector construction
NtHSR201-F	CAGCAGTCCTTTGGCGTTGTC	qRT-PCR
NtHSR201-R	GCTCAGTTTAGCCGCAGTTGTG	qRT-PCR
NtHSR203-F	TGGCTCAACGATTACGCA	qRT-PCR
NtHSR203-R	GCACGAAACCTGGATGG	qRT-PCR
NtHSR51-F	TTGGGCAGAATAGATGGGTA	qRT-PCR
NtHSR51-R	TTTGGTGAAAGTCTTGGCTC	qRT-PCR
NtNPR1-F	GGCGAGGAGTCCGTTCTTTAA	qRT-PCR
NtNPR1-R	TCAACCAGGAATGCCACAGC	qRT-PCR
NtPR-1a/c-F	AACCTTTGACCTGGGACGAC	qRT-PCR
NtPR-1a/c-R	GCACATCCAACACGAACCGA	qRT-PCR
NtPR2-F	TGATGCCCTTTTGGATTCTATG	qRT-PCR
NtPR2-R	AGTTCCTGCCCCGCTTT	qRT-PCR
NtPR3-F	CAGGAGGGTATTGCTTTGTTAGG	qRT-PCR
NtPR3-R	CGTGGGAAGATGGCTTGTTGTC	qRT-PCR
NtEFE26-F	CGGACGCTGGTGGCATAAT	qRT-PCR
NtEFE26-R	CAACAAGAGCTGGTGCTGGATA	qRT-PCR
NtAccdeaminase-F	TCTGAGGTTACTGATTTGGATTGG	qRT-PCR
NtAccdeaminase-R	TGGACATGGTGGATAGTTGCT	qRT-PCR
NtEF1-α-F	TGCTGCTGTAACAAGATGGATGC	qRT-PCR
NtEF1-α-R	GAGATGGGGACAAAGGGGATT	qRT-PCR

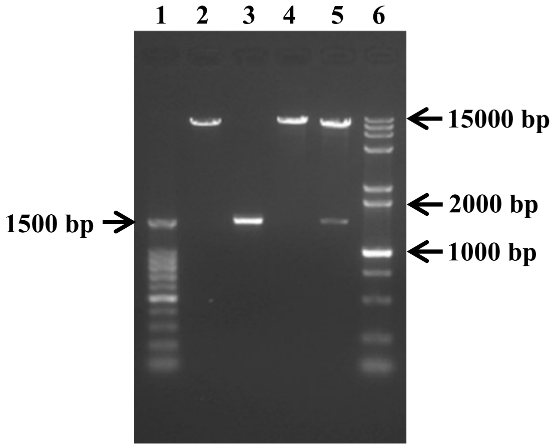

Figure S1. Enzyme digestion of the subcellular localization expression vector pCAMBIA 2300-*ScCAT2-GFP*. 1, 100 bp ladder marker; 2, *35S::GFP/Xba*I; 3, *ScCAT2* ORF PCR product; 4, *35S::ScCAT2::GFP/Xba*I; 5, *35S::ScCAT2::GFP/Xba*I + *Spe*I; 6, 15000 + 2000 bp DNA marker.

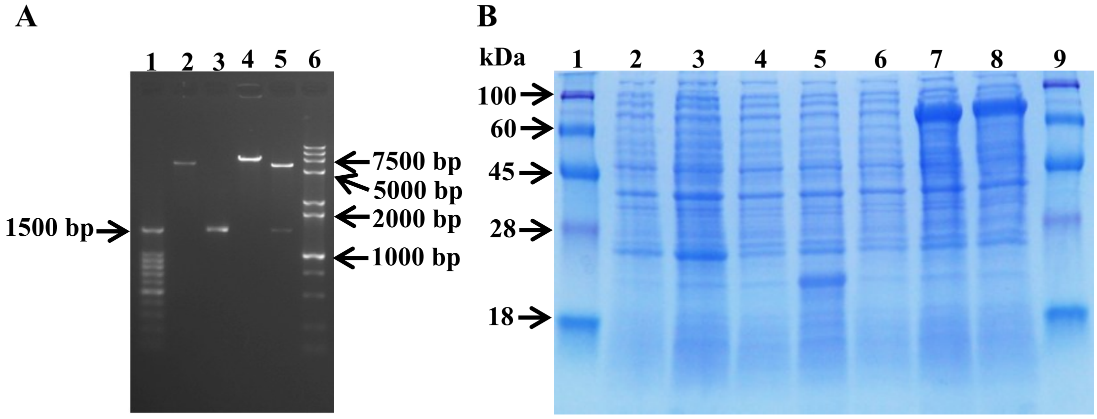

Figure S2. Enzyme digestion of prokaryotic expression vector pET-32a-*ScCAT2* (A) and corresponding protein expression in *Escherichia coli* Rosetta strain cells (B). (A) 1, 100 bp ladder marker; 2, pET-32a/*Eco*RI; 3, *ScCAT2* ORF PCR product; 4, pET-32a-*ScCAT2*/*Eco*RI; 5, pET-32a-*ScCAT2*/*Eco*RI + *Xho*I; 6, 15000 + 2000 bp DNA marker. (B) 1, Protein marker; 2, Rosetta strain cells without induction; 3, Rosetta strain cells induction for 2 h; 4, Rosetta + pET-32a strains without induction; 5, Rosetta + pET-32a strains induction for 2 h; 6, Rosetta + pET-32a-*ScCAT2* strains without induction; 7 and 8, Rosetta + pET-32a-*ScCAT2* strains induction for 1 h and 2 h, respectively.

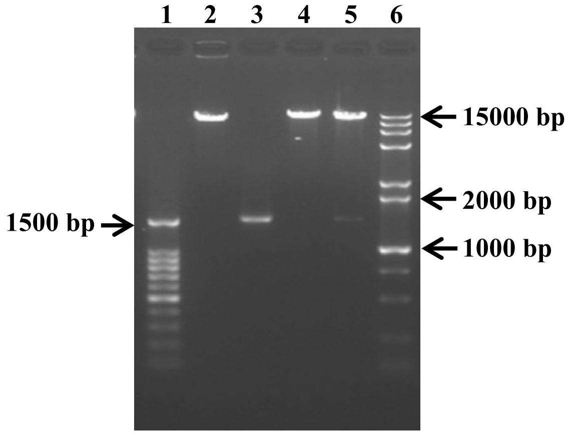

Figure S3. Enzyme digestion of the transient overexpression vector pCAMBIA 1301-*ScCAT2*. 1, 100 bp ladder marker; 2, pCAMBIA 1301/*Xba*I; 3, *ScCAT2* ORF PCR product; 4, pCAMBIA 1301-*ScCAT2*/*Xba*I; 5, pCAMBIA 1301-*ScCAT2*/*Xba*I + *Spe*I; 6, 15000 + 2000 bp DNA marker.
